# Data set from the phosphoproteomic analysis of *Magnaporthe oryzae*-responsive proteins in susceptible and resistant rice cultivars

**DOI:** 10.1016/j.dib.2014.12.009

**Published:** 2015-01-27

**Authors:** Yunfeng Li, Zhijian Ye, Yanfang Nie, Jian Zhang, Guo-Liang Wang, Zhenzhong Wang

**Affiliations:** a Laboratory of Physiological Plant Pathology, South China Agricultural University, Guangzhou 510642, China; b Guangdong Province Key Laboratory of Microbial Signals and Disease Control, South China Agricultural University, Guangzhou 510642, China; c College of Natural Resources and Environment, South China Agricultural University, Guangzhou 510642, China; d Department of Plant Pathology, Ohio State University, Columbus 43210, OH, USA

## Abstract

Rice blast, caused by the fungal pathogen *Magnaporthe oryzae*, is the most destructive disease of rice and causes tremendous losses of rice yield worldwide. To explore the molecular mechanisms involved in the rice–*M. oryzae* interaction, we conducted a time-course phosphoproteomic analysis of leaf samples from resistant and susceptible rice cultivars infected with *M. oryzae*. This data article contains additional results and analysis of *M. oryzae*-regulated phosphoproteins in rice leaves [1]. We report the analysis of *M. oryzae*-regulated phosphoproteins at all time points, including Venn diagram analysis, close-up views, relative intensities, and functional category, and the MS spectra of representative phosphoprotein and representative phosphorylated peptides.

**Table:** **Specifications table**.

Subject area	Biology
More specific subject area	Plant proteomics
Type of data	Table, figure
How data was acquired	Mass spectroscopy, data acquired using ABI 4800 Proteomics Analyzer (Applied Biosystems)
Data format	Analyzed
Experimental factors	Rice leaf phosphoproteins were enriched using a combination of PEG-mediated protein prefractionation technique and Al(OH)_3_-MOAC method. The phosphoprotein was re-dissolved in buffer solution containing 7 M urea, 2 M thiourea, 2% v/v NP-40, 65 mM DTT, and 1% IPG buffer. Protein content was determined by Bradford assay using BSA as a standard.
Experimental features	The 2-D gels were stained with Pro-Q diamond phosphoprotein stain. MS and MS/MS spectra were obtained using the ABI 4800 Proteomics Analyzer MALDI-TOF/TOF.
Data source location	South China Agricultural University, Guangzhou, China
Data accessibility	Data is supplied with this article

Value of the data

• First phosphoproteome analysis of rice leaves after *M. oryzae* infection
• Quantitative analysis revealed 56 *M. oryzae*-regulated phosphoproteins.
• Phosphorylation sites within 7 identified phosphoproteins was verified by NanoLC–MS/MS

## 1 Data, experimental design, materials and methods

A time-course phosphoproteomic analysis of rice leaves was performed after *M. oryzae* inoculation, including venn diagram analysis of the phosphoproteins overlapped between two cultivars (Supplementary Material, Fig. 1), close-up views of the phosphoprotein spots in 2-DE gels (Supplementary Material, Fig. 2), relative intensities of all phosphoprotein spots (Supplementary Material, Fig. 3), the MS spectra of representative phosphoprotein (Supplementary Material, Fig. 4), functional category (Supplementary Material, Fig. 5), and the MS/MS spectra of representative phosphorylated peptides (Supplementary Material, Fig. 6). A detailed experimental procedure was generated for “identification of phosphorylation sites by NanoLC–MS/MS”. Furthermore, gene-specific primer sequences were designed for qRT-PCR (Supplementary Material, Table 1).

## 2 Materials

Two near isogenic lines of rice (*Oryza sativa indica*), C101LAC and CO39, were provided by the International Rice Research Institute IRRI, Philippines. C101LAC contains the resistance gene *Pi-1* in the CO39 background. The *Magnaporthe oryzae* race ZC_13_, one of the primary *M. oryzae* races in Guangdong Province, is incompatible with C101LAC but compatible with CO39 [2]. Maintenance of fungal cultures, spore solution preparation, and growth condition of rice plants were as previously [3]. Rice seedlings at the four-leaf stage were spray-inoculated with freshly prepared *M. oryzae* spores (1×10^5^ conidia/mL, containing 0.02% v/v Tween 20). The fourth leaves were harvested at 8, 12, and 24 h post-inoculation for protein extraction and 2-DE analysis. The leaf samples were frozen in liquid nitrogen and stored at −80 °C. Control plants were sprayed with sterilized water including 0.02% v/v Tween 20.

## 3 Preparation of samples for proteomics analysis

Total proteins were extracted essentially as previously described with some modifications [4]. Briefly, a 5-g quantity of leaf tissue of each sample was extracted with Mg/NP-40 buffer (containing 0.5 M Tris–HCl, pH 8.8, 2% v/v NP-40, 20 mM MgCl_2_, 2% v/v 2-mercaptoethanol, 1% w/v PVPP, 1 mM PMSF, 40 mM NaF, and 15 μL of cocktail) and fractionated with PEG 4000. Protein was precipitated with four volumes of ice-cold acetone for 2 h at −20 °C and dissolved in incubation buffer (IB) (30 mM MES, 30 mM imidazole, 8 M urea, 0.1 M potassium aspartate, 0.1 M sodium glutamate, and 0.25% w/v CHAPS). Protein concentration was determined using the procedure as described previously, with BSA as the standard [5]. Each experiment was repeated three times with different pools of leaf samples.

## 4 Enrichment of phosphoproteins using Al(OH)_3_-MOAC

Phosphorylated proteins were enriched from the total protein extract using the Al(OH)_3_-MOAC method as previously described with modifications [6]. Approximately 8 mg of total protein extract was added to 2 g of Al(OH)_3_. After centrifugation (6000*g* for 10 min), the matrix with retained proteins was washed four times with the wash buffer (similar to IB except for the amino acid concentration was 0.15 M). The bound putative phosphoproteins were then eluted with 20 mL of elution buffer (0.3 M potassium pyrophosphate, 8 M urea, pH 9). After centrifugation (10,000*g* for 5 min), the supernatant was removed from the matrix and recentrifuged to avoid matrix carryover. The eluted proteins were amended with 2% w/v sodium deoxycholate and 100% w/v trichloroacetic acid (TCA) and placed at 4 °C overnight. After centrifugation (18,000*g* for 15 min), precipitated proteins were successively collected and washed with 25% w/v TCA. The pellets were then resuspended in 10 mM Tris–HCl (pH 7.5) in 80% v/v acetone and washed with 90% v/v acetone after centrifugation. After air drying, the phosphoproteins were dissolved in resolubilization/rehydration buffer (RB) (7 M urea, 2 M thiourea, 2% v/v NP-40, 65 mM DTT, and 1% IPG buffer).

## 5 Gel electrophoresis and staining of gels

Two-dimensional gel electrophoresis (2-DE) was performed as described previously [7]. Briefly, gels were loaded with 150 μg of proteins in 350 μL RB buffer. Isoelectric focusing (IEF) was performed in a stepwise manner: at 100 V for 0.5 h, 500 V for 0.5 h, 1000 V for 2 h, 8000 V gradients for 4 h and 8000 V for total of 60,000 V h. SDS-PAGE in the second dimension was then conducted with 12% gels. For phosphoproteins detection, 2-D gels were stained with a modified protocol using Pro-Q Diamond fluorescent gel stain [8]; total proteins were stained with the improved Coomassie brilliant blue G-250 (CBB) staining method [9]. Briefly gels were treated with fixation solution (50% methanol, 10% acetic acid) (2×30 min), washed with deionized water (3×10 min), stained with 3-fold diluted Pro-Q DPS in deionized water (2 h), destained with destaining solution (20% ACN, 50 mM sodium acetate pH 4.0) (4×30 min) to remove gel-bound nonspecific Pro-Q DPS, and washed again with deionized water (2×5 min). The gel was then scanned with a Typhoon Trio Variable Mode Imager (GE Healthcare, Uppsala Sweden) with a 532 nm laser excitation and a 580 nm bandpass emission filter. Spot identification, background elimination, point matching, and differential analysis of the protein spots were completed using PDQuest software (Version 8.0, Bio-Rad, Hercules, CA, USA). The proteins spots showing ≥2-fold changes in all three biological replicas were considered as differentially regulated putative phosphoproteins.

## 6 MALDI-TOF/TOF MS analysis and database searching

Selected protein spots were excised from the CBB-stained gels, and then destained with 100 mM NH_4_HCO_3_ in 30% acetonitrile (ACN). After removing the destaining buffer, the gel pieces were lyophilized and rehydrated in 30 μL of 50 mM NH_4_HCO_3_ containing 50 ng trypsin. After overnight digestion at 37 °C, the peptides were extracted three times with 0.1% TFA in 60% ACN. Extracts were pooled together and lyophilized. A protein-free gel piece was treated as above and used as a control to identify autoproteolysis products derived from trypsin. MS and MS/MS spectra were obtained using the ABI 4800 Proteomics Analyzer MALDI-TOF/TOF (Applied Biosystems, Foster City, CA). Peptide mass maps were acquired in positive ion reflector mode with 1000 laser shots per spectrum. Monoisotopic peak masses were automatically determined within the mass range 800–4000 Da with a signal to noise ratio minimum set to10 and a local noise window width of *m*/*z* 250. After excluding common trypsin autolysis peaks and matrix ion signals, up to five most intense ions with a minimum signal to noise ratio higher than 50 were selected as candidates for MS/MS acquisition. In MS/MS-positive ion mode, spectra were averaged, collision energy was set at 2 kV, and default calibration was selected. Monoisotopic peak masses were automatically determined with a signal to noise ratio minimum set to 5 and a local noise window width of *m*/*z* 250. The MS together with MS/MS spectra were searched against the UniprotKB/SwissProt database (v.2013.01.24) using the software GPS Explorer version 3.6 (Applied Biosystems) and MASCOT version 2.2 (Matrix Science, London, UK) with the following parameter settings: trypsin cleavage, one missed cleavage allowed, carbamidomethylation set as fixed modification, peptide mass tolerance set to 100 ppm, fragment tolerance set to ±0.3 Da, and minimum ion score confidence interval for MS/MS data set to 95%.

## 7 Identification of phosphorylation sites by NanoLC–MS/MS

To determine phosphorylation sites of the identified phosphoprotein, we isolated total proteins from C101LAC and CO39 at 12 h post-inoculation and mixed them in a 1:1 (w/w total protein) ratio. Protein digestion was performed according to the FASP procedure as previously described [10]. Briefly, 1 mg mixed phosphoproteins were reduced and alkylated by successive incubation in 100 mM DTT and 50 mM iodoacetamide, following digestion with 2 μg trypsin overnight. Then, the peptide mixture was loaded onto aliquot of titanium dioxide (TiO_2_) beads twice. The two eluted fractions were subjected to MS analysis. NanoLC–MS/MS was performed with a Q Exactive MS (Thermo Finnigan) equipped with Easy nLC (ThermoFisher, San Jose, CA). The peptide mixture was loaded onto a C18-reversed phase column with a flow rate of 250 nL/min over 240 min. For MS/MS analysis, peptides were analyzed in positive ion mode. MS/MS spectra were searched using Mascot 2.2 engine against the Uniprot_Oryza database (v.2013.01.24) and the reversed database. The phosphorylation peptides were analyzed using Proteome Discoverer 1.3 (Thermo Electron, San Jose, CA). pRS score above 50 indicate a good PSM (Peptide Spectrum Matches) and pRS probabilities above 75% indicate that a site is truly phosphorylated.

## 8 Quantitative real-time PCR (qRT-PCR) analysis

Total RNA was isolated from rice leaves using the LiCl density gradient method [11]. Gene-specific primers were designed based on the nine *M. oryzae*-regulated gene sequences using the Primer 3.0 software; the primer sequences are listed in Supplementary Material, Table 1. The tubulin gene (GenBank accession no. BAA02241) was designated as a reference gene. qRT-PCR was carried out with the SYBR Green I PCR Master Mix (Takara) according to the manufacturer׳s protocol, and all qRT-PCR reactions were performed using the Thermal Cycler Dice (Takara, Otsu, Shiga, Japan). RT-PCR conditions were as follows: 95 °C for 30 s, followed by 40 cycles of 95 °C for 5 s and 60 °C for 30 s. Each sample was represented by three independent biological replicates, and each biological replicate was analyzed in three technical replicates. The relative changes in gene expression levels were calculated using the 2^−ΔΔCt^ method [12].

## 9 Statistical analysis

Statistical analysis was carried out using SPSS software (Release v14.0, SPSS Company, USA). Analysis of covariance (ANCOVA) followed by Duncan׳s multiple range test was used for analysis of the *M. oryzae*-regulated proteins and qRT-PCR. The data are presented as means±SE (standard error).

## Conflict of interests

The authors declare that they have no competing interests.
