# Supplementary material for: Data set from the phosphoproteomic analysis of Magnaporthe oryzae-responsive proteins in susceptible and resistant rice cultivars
Source: Data Brief. 2015 Jan 27;3:7–11. doi: 10.1016/j.dib.2014.12.009 (PMC4509991; doi:10.1016/j.dib.2014.12.009)
Supplement: Supplementary file 1 — Supplementary data [file mmc1.pdf]

Data in Brief

Data set from the phosphoproteomic analysis of *Magnaporthe oryzae* -responsive proteins in susceptible and resistant rice cultivars

Yunfeng Li, Zhijian Ye, Yanfang Nie, Jian Zhang, Guo-Liang Wang, and Zhenzhong Wang

Figure 1

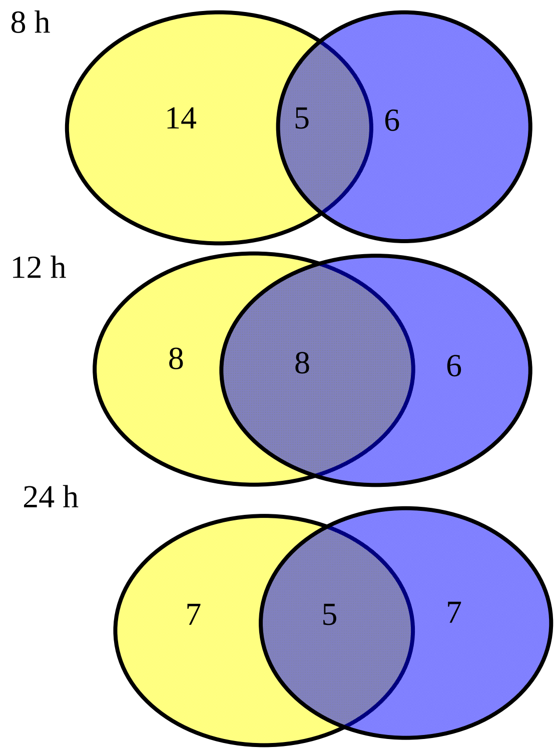

Figure 1. Venn diagram analysis of the *M. oryzae*-regulated phosphoproteins that overlapped between the susceptible cultivar CO39 (yellow) and resistant cultivar C101LAC (blue).

Rice plants were treated with *M. oryzae* for a time course. In total, 56 phosphoproteins were found to be regulated by *M. oryzae*. The diagram shows the number of proteins that are phosphorylated specifically in each cultivar as well the number phosphorylated in both cultivars.

Figure 2

Figure 2. Close-up views of the regions of 2-DE gels showing all *M. oryzae*-regulated phosphoprotein spots in two rice cultivars.

Arrows indicate phosphoproteins whose expression changed in response to fungal inoculation. The relative locations of these protein spots are indicated in Figure 1. Symbol: CK, rice control treated with water; *M. oryzae*, rice inoculated with *M. oryzae*.

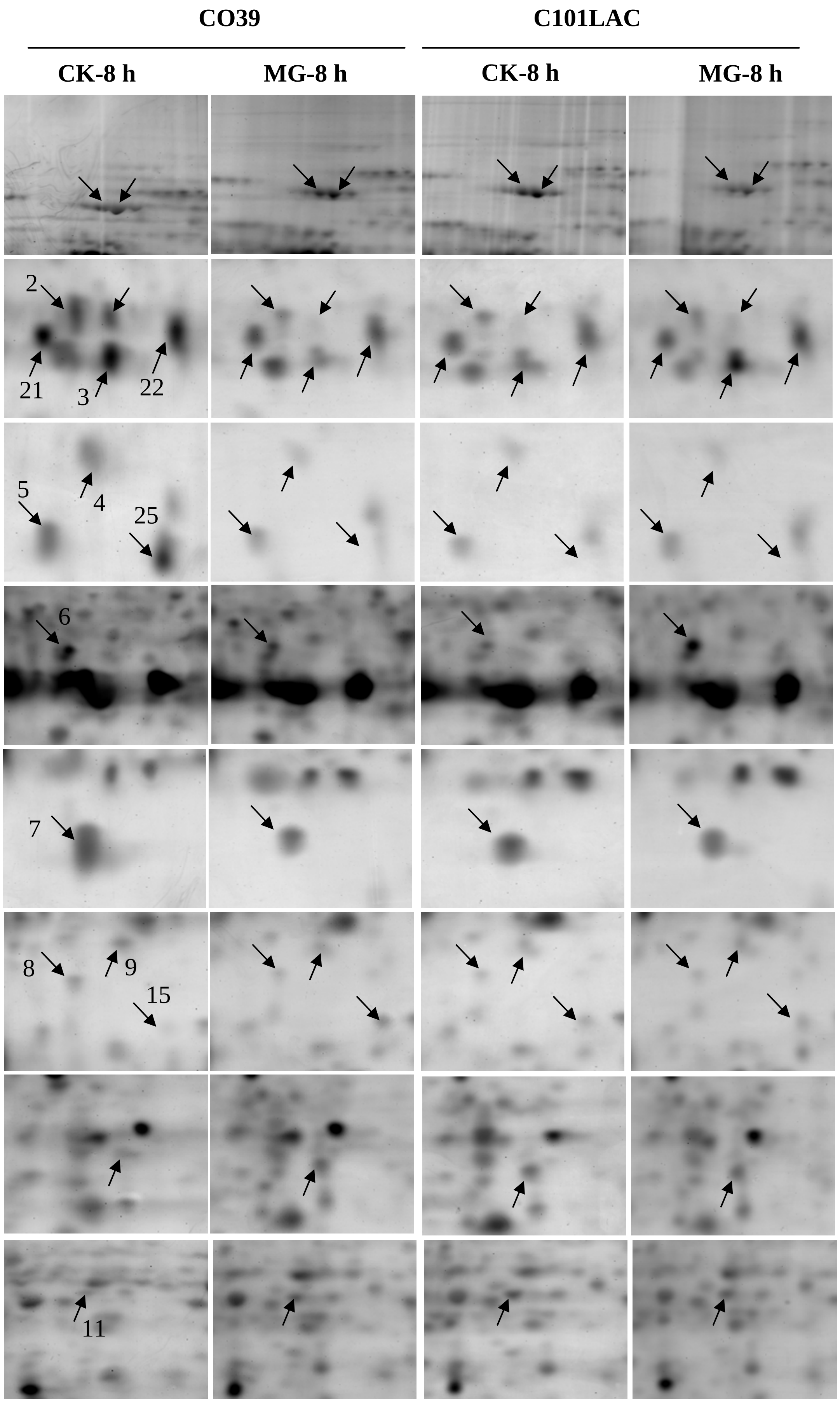

CO39
C101LAC
CK-8 h
MG-8 h
CK-8 h
MG-8 h
2
21
3
22
5
4
25
6
7
8
9
15
11

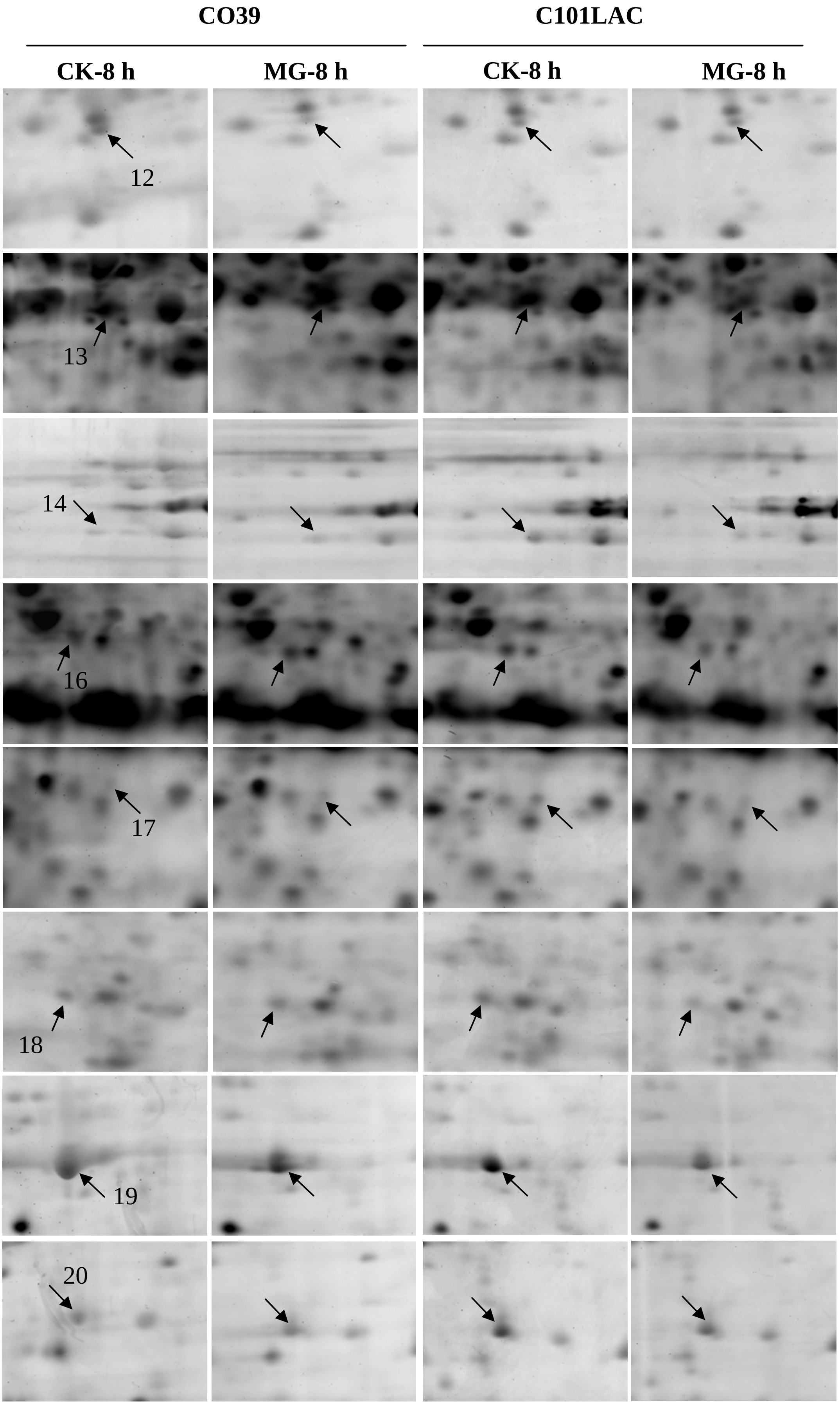
CO39
C101LAC
CK-8 h
MG-8 h
CK-8 h
MG-8 h
12
13
14
16
17
18
19
20

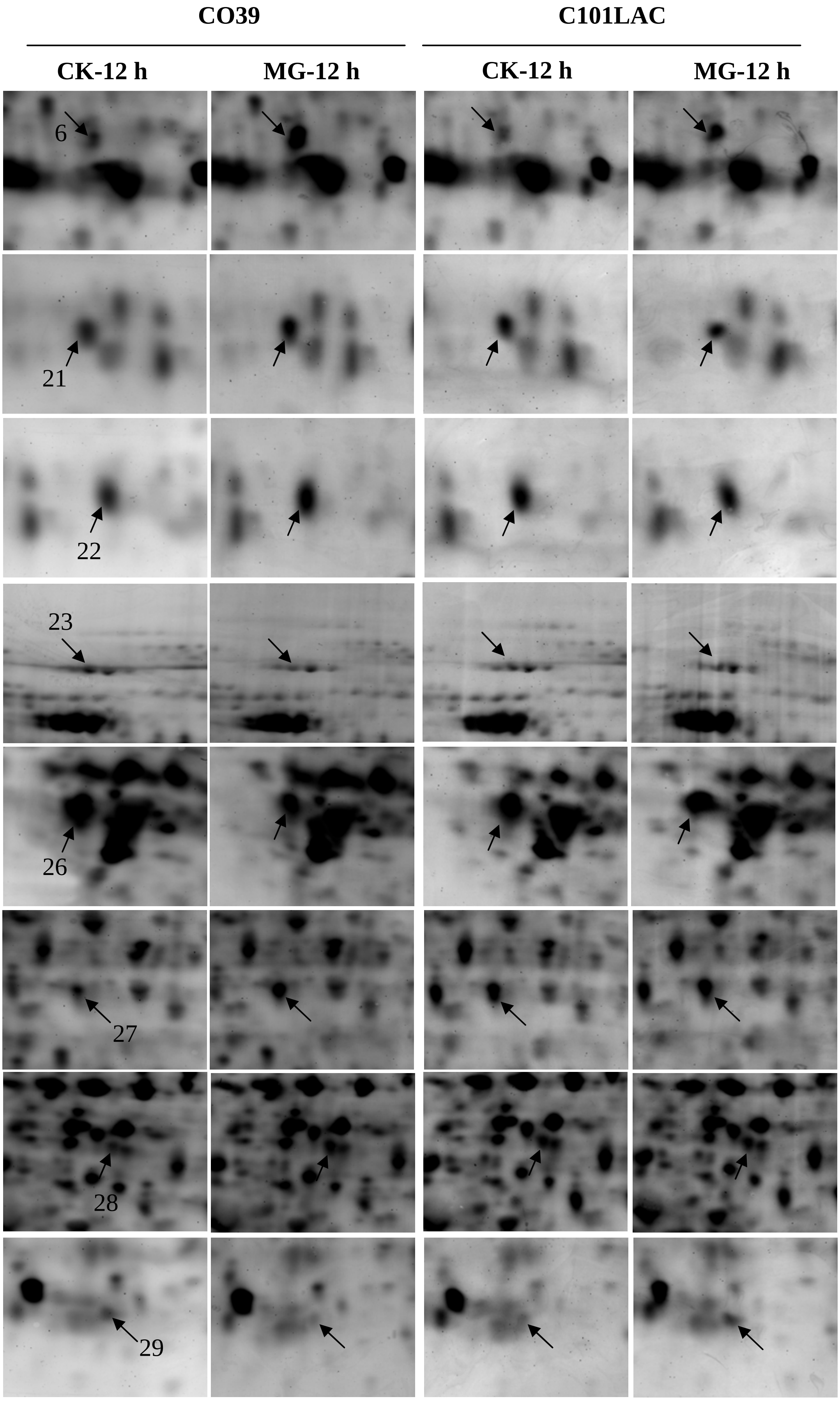
CO39
C101LAC
CK-12 h
MG-12 h
CK-12 h
MG-12 h
6
21
22
23
26
27
28
29

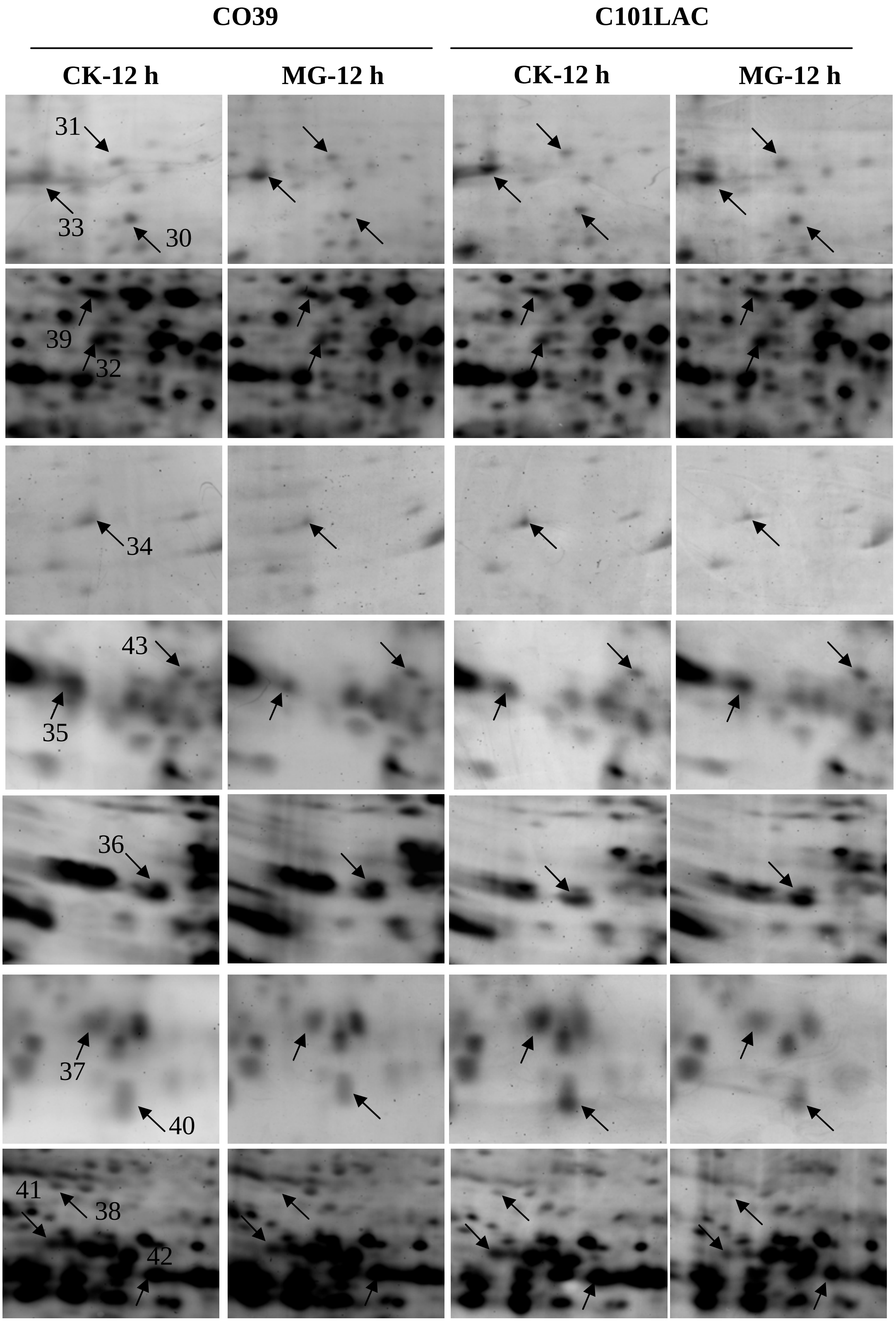

CO39
C101LAC
CK-12 h
MG-12 h
CK-12 h
MG-12 h
31
33
30
39
32
34
43
35
36
37
40
41
38
42

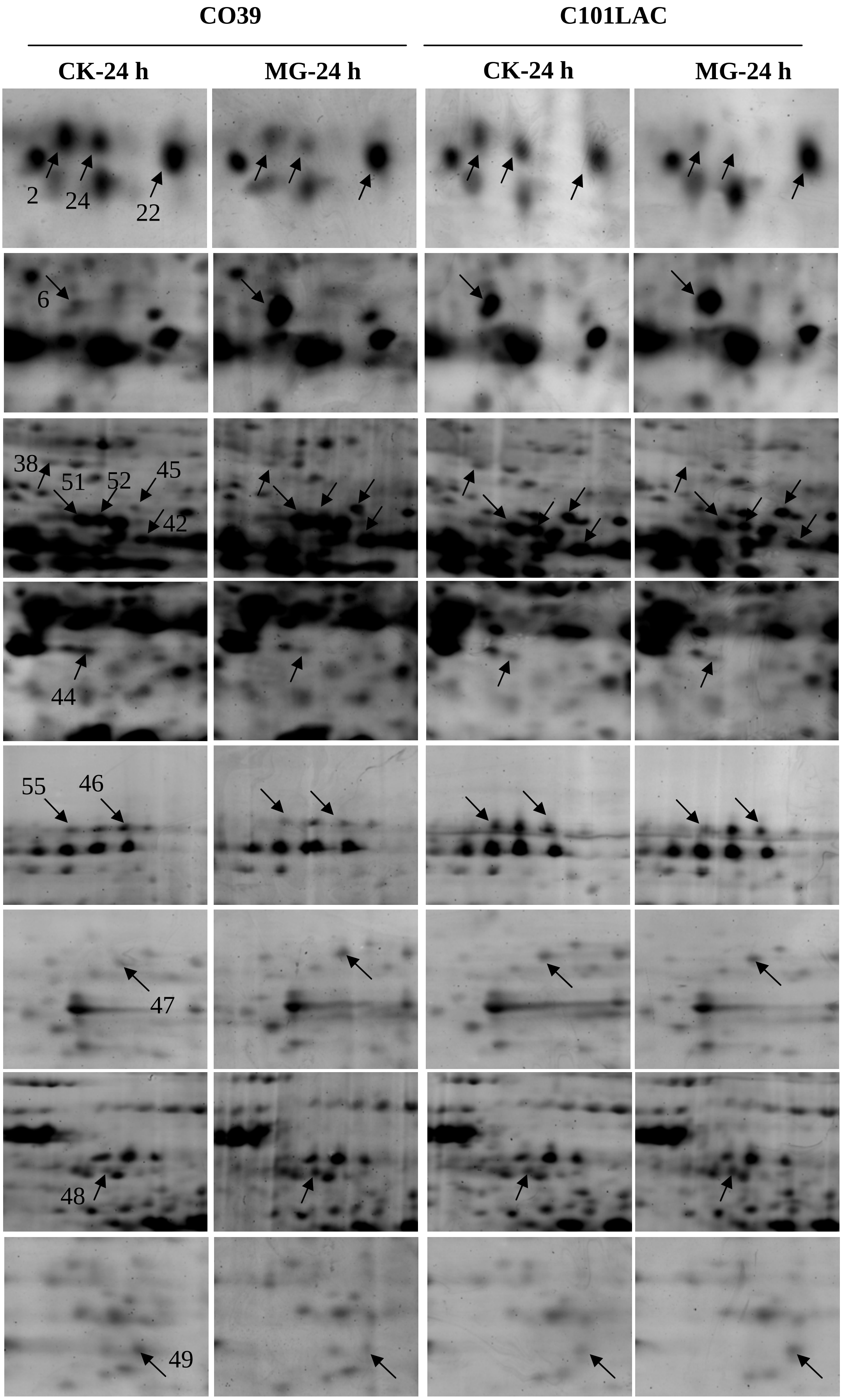
CO39
C101LAC
CK-24 h
MG-24 h
CK-24 h
MG-24 h
2
24
22
6
38
51
52
45
42
44
55
46
47
48
49

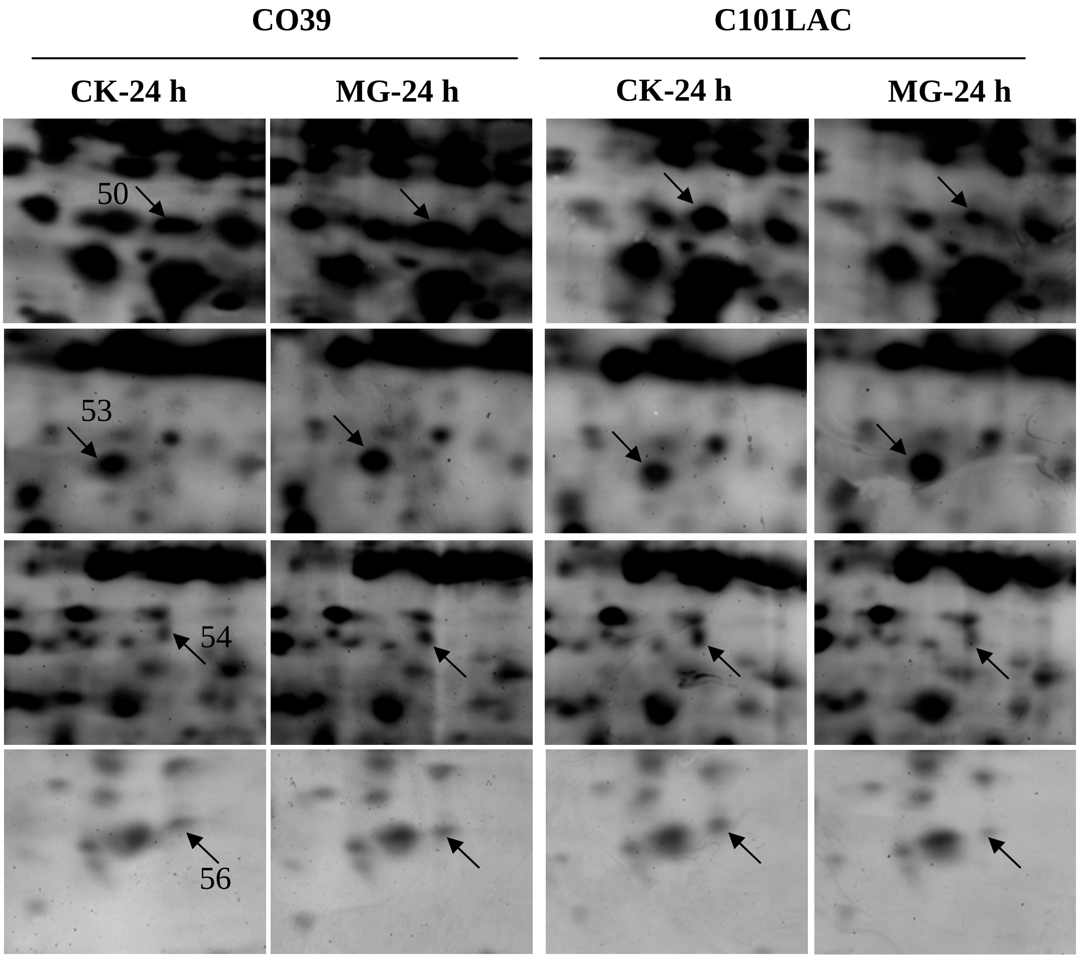
CO39
C101LAC
CK-24 h
MG-24 h
CK-24 h
MG-24 h
50
53
54
56

Figure 3

Figure 3. Quantitative analysis of the *M. oryzae*-regulated phosphoproteins in rice leaves.

At 8 h (A), 12 h (B), and 24 h (C) after *M. oryzae* inoculation, rice leaf phosphoprotein samples were prepared and separated by 2DE. The protein intensity of the protein spots were calculated with PDQuest 8.0 software. Values are the means (±SE) of protein intensity on gels from three independent experiments. Symbol: CK, rice control treated with water; *M. oryzae*, rice inoculated with *M. oryzae*.

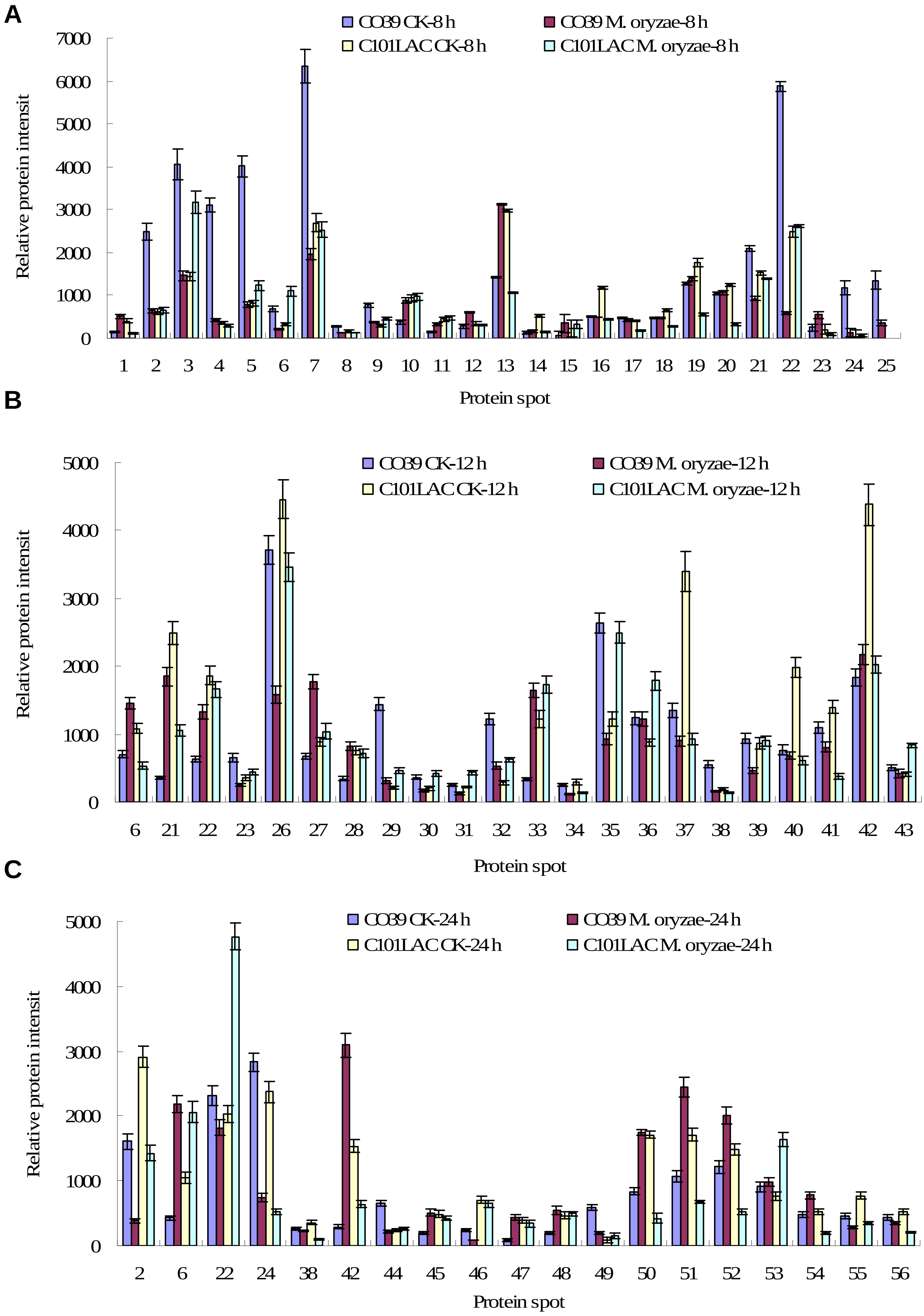
A
CO39 CK-8 h
CO39 M. oryzae-8 h
C101LAC CK-8 h
C101LAC M. oryzae-8 h
Relative protein intensit
Protein spot
B
CO39 CK-12 h
CO39 M. oryzae-12 h
C101LAC CK-12 h
C101LAC M. oryzae-12 h
Relative protein intensit
Protein spot
C
CO39 CK-24 h
CO39 M. oryzae-24 h
C101LAC CK-24 h
C101LAC M. oryzae-24 h
Relative protein intensit
Protein spot

Figure 4

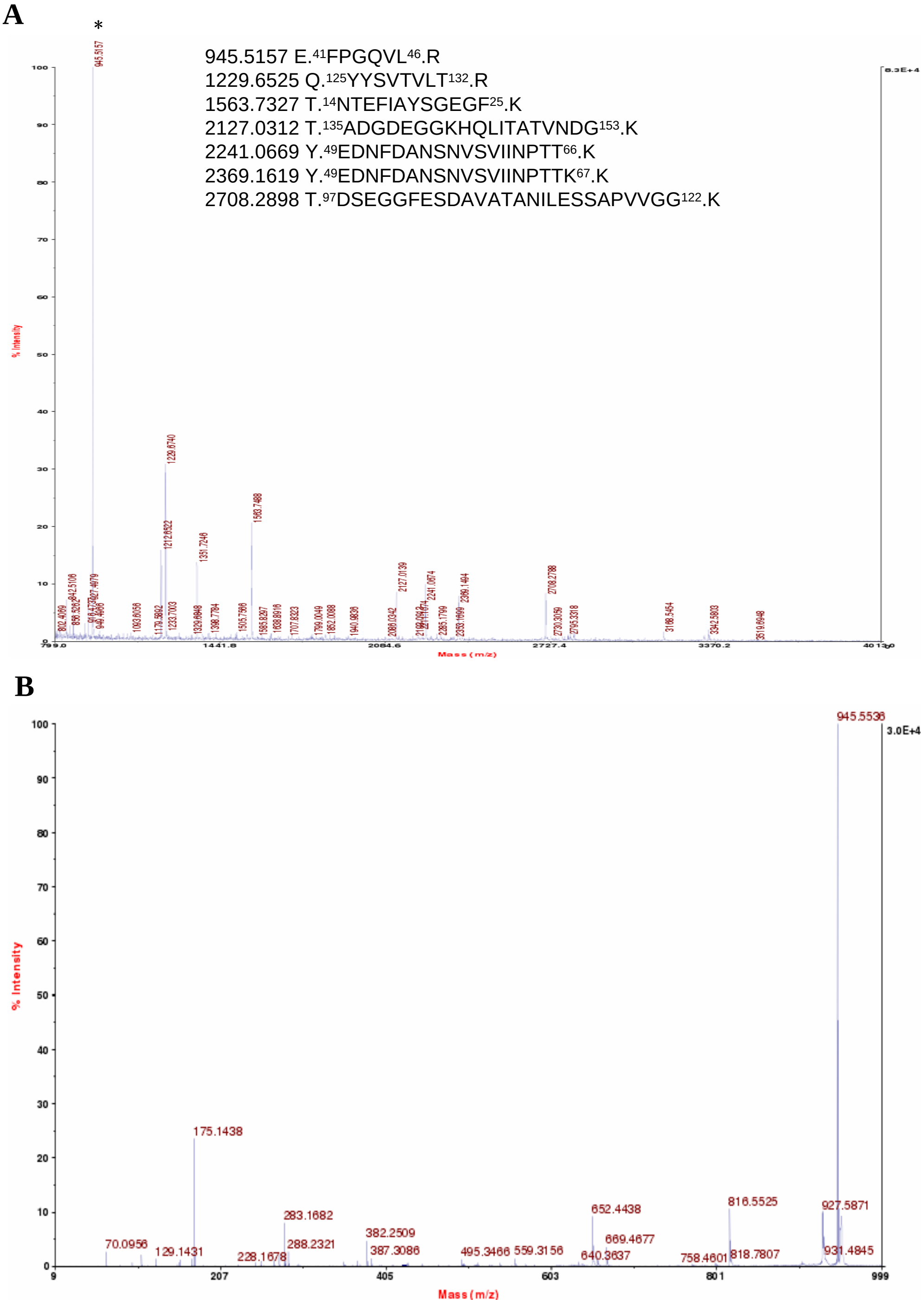

Figure 4. Identification of spot 4 by MALDI-TOF/TOF MS.

The protein excised from CBB-staining gels was digested with trypsin, and the resulting peptides were analyzed using the 4800 Proteomic Analyzer. A, The MS spectra. The matched peptides and their corresponding peaks are listed in the map. The ion 945.5 marked with an asterisk was analyzed by MS/MS. B, MS/MS spectra of ion 945.5. The corresponding peptide sequence is shown. After database searching, the protein was identified as Chloroplast 23 kDa polypeptide of photosystem II.

Figure 5

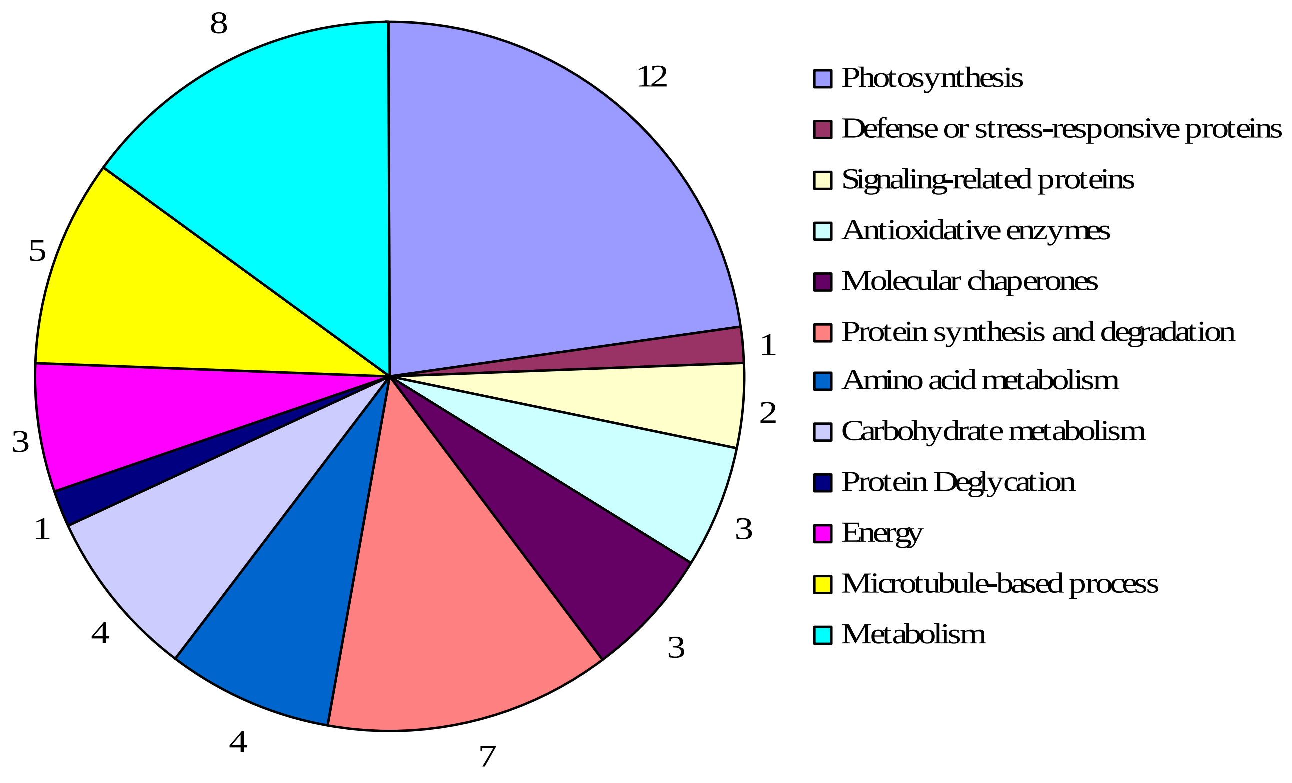

Figure 5. The functional category distribution of the 53 *M. oryzae*-regulated phosphoproteins.

Figure 6

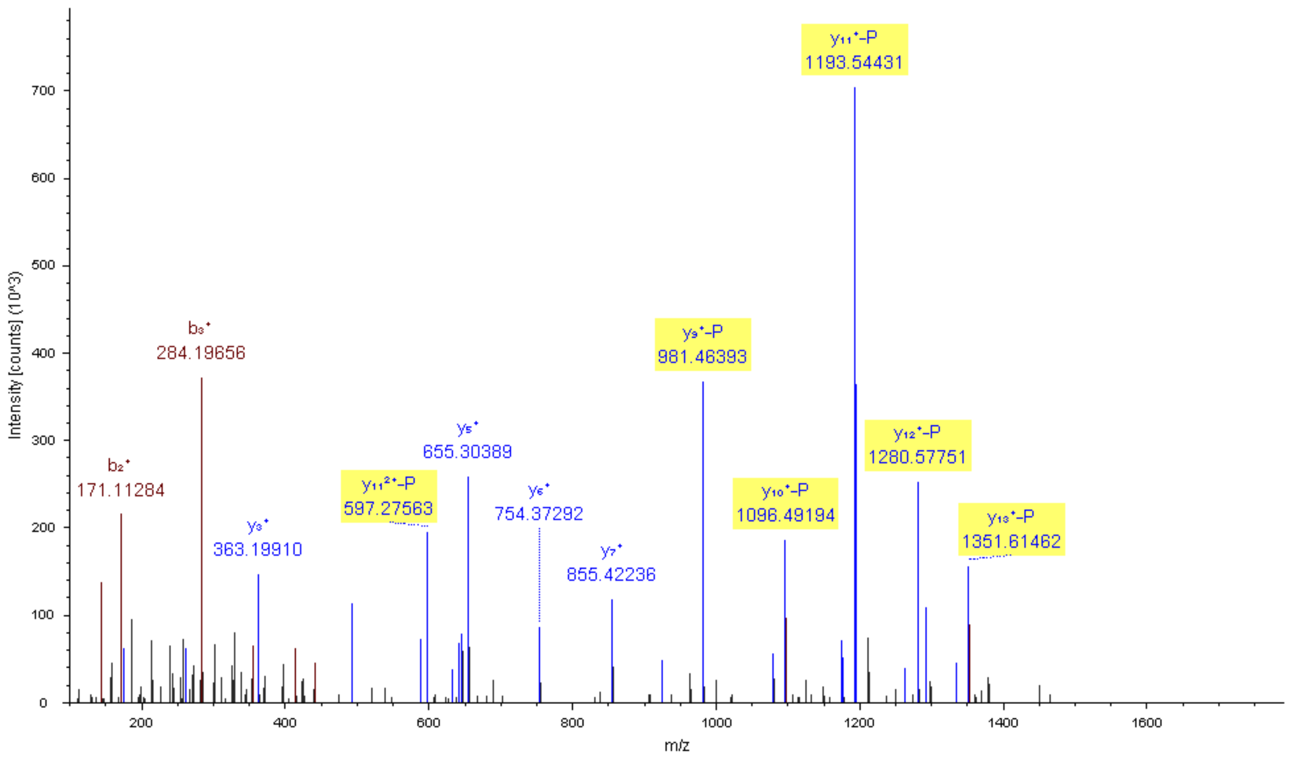

Figure 6. The MS/MS spectra of representative phosphorylated peptides of GLLASPDG*TVYETSR, corresponding to porphobilinogen deaminase, chloroplastic (Q6H6D2).

The asterisk indicates phosphorylation on the right side of the serine residue.
