# Supplementary material for: Data set from the phosphoproteomic analysis of Magnaporthe oryzae-responsive proteins in susceptible and resistant rice cultivars
Source: Data Brief. 2015 Jan 27;3:7–11. doi: 10.1016/j.dib.2014.12.009 (PMC4509991; doi:10.1016/j.dib.2014.12.009)
Supplement: Supplementary file 2 — Supplementary data [file mmc2.doc]

**Experimental Procedures**

**Identification of phosphorylation sites by NanoLC-MS/MS**

**(1) Protein digestion**

To determine phosphorylation sites of the identified phosphoprotein, respective MOAC-enriched phosphoproteins samples from C101LAC and CO39 at 12 h post-inoculation were selected and mixed in a 1:1 (w/w total phosphoprotein) ratio. Phosphoprotein digestion was performed according to the FASP procedure as previously described (Wiśniewski et al, 2009). Briefly, 1 mg proteins for each sample were incorporated into 30 μL STD buffer (4% SDS, 100 mM DTT, 150 mM Tris-HCl, pH 8.0). The detergent, DTT and other low-molecular-weight components were removed using UA buffer (8 M Urea, 150 mM Tris-HCl pH 8.0) by repeated ultrafiltration (Microcon units, 30 kD). Then 100 μl 0.05 M iodoacetamide in UA buffer was added to block reduced cysteine residues and the samples were incubated for 20 min in darkness. The filters were washed with 100 μl UA buffer three times and then 100 μl 25 mM NH_4_HCO_3_ (pH 8.5) twice. Finally, the protein suspensions were digested with 2 μg trypsin (Promega) in 40 μl 25 mM NH_4_HCO_3_ overnight at 37 °C, and the resulting peptides were collected as a filtrate.

**(2) Enrichment of phosphorylated peptiedes by the TiO_2_ beads**

The final in-solution digested peptide mixture was concentrated by a vacuum concentrator and resuspended in 500 µL loading buffer (2% glutamic acid/ 65% ACN/ 2% TFA). Then, TiO_2_ beads (5 μm Titansphere, GL Sciences, Japan) were added and then agitated for 40 min. The centrifugation was carried out for 1 min at 5000 g, resulting the first beads. The supernatant from the first centrifugation were mixed with another TiO_2_ beads, resulting in the second beads which collected as before. Both beads were combined and washed with 50 uL of washing buffer I (30% ACN/ 3%TFA) three times and then 50 uL of washing buffer II (80% ACN/0.3% TFA) three times to remove the remaining non-adsorbed material. Finally, the phosphopeptides were eluted with 50 uL of elution buffer (40% ACN/15% NH_4_OH)^[3]^, followed by lyophilization and MS analysis.

**(3) Nano-LC-MS System**

5ul of the phosphopeptides solution mixed with 15ul 0.1% (v/v) trifluoroacetic acid and then 10ul of the solution mixture was injected for nanoLC-MS/MS analysis using an Q Exactive MS (Thermo Finnigan) equipped with Easy nLC (ThermoFisher, San Jose, CA). The peptide mixture was loaded onto a C18-reversed phase column (15 cm long, 75μm inner diameter, RP-C18 3μm, packed in-house) in buffer A (0.1% Formic acid) and separated with a linear gradient of buffer B (80% acetonitrile and 0.1% Formic acid) at a flow rate of 250nL/min controlled by IntelliFlow technology over 240 min. The peptides were eluted with a gradient of 0%–60% buffer B from 0 min to 200min, 60% to 100% buffer B from 200min to 216 min, 100% buffer B from 216min to 240min.

For MS analysis, peptides were analyzed in positive ion mode. MS spectra were acquired using a data-dependent top10 method dynamically choosing the most abundant precursor ions from the survey scan (300–1800m/z) for HCD fragmentation. Determination of the target value is based on predictive Automatic Gain Control (pAGC). Dynamic exclusion duration was 40.0s. Survey scans were acquired at a resolution of 70,000 at m/z 200 and resolution for HCD spectra was set to 17,500 at m/z 200. Normalized collision energy was 27eV and the under fill ratio, which specifies the minimum percentage of the target value likely to be reached at maximum fill time, was defined as 0.1%. The instrument was run with peptide recognition mode enabled.

**(4) Data Analysis**

MS/MS spectra were searched using Mascot 2.2 engine against the Uniprot_Oryza database (212787 sequences, download Jan 24^th^, 2013) and the reversed database. For protein identification, the following options were used. Peptide mass tolerance: 20 ppm; MS/MS tolerance: 0.1 Da; enzyme: trypsin: missed cleavage: 2; fixed modification: carbamidomethyl (C); variable modification: oxidation(M); phosphorylation: S/T/Y; FDR (false discovery rates) ≤0.01. The phosphorylation peptides were analyzed using Proteome Discoverer 1.3 (Thermo Electron, San Jose, CA.). pRS score above 50 indicate a good PSM (Peptide Spectrum Matches) and pRS probabilities above 75 percent indicate that a site is truly phosphorylated.

**Table 1. Gene-specific primer sequences designed for qRT-PCR**

| Spot no. | Protein name | NCBI accession No. | Forward primer (5’→3’) | Reverse primer (5’→3’) | Expected fragment (bp) |
| --- | --- | --- | --- | --- | --- |
| 3 | L-ascorbate peroxidase 1, cytosolic | gi|115452337 | CCCTCTCTGGCGGTCACA | CCCTCAAAACCAGATCTTTCCTT | 58 |
| 9 | Protein-ribulosamine 3-kinase, chloroplastic | gi|357580450 | GGGCTATGGTTTCTATGTTGACAA | CGGCAGTCCAAGTGTTAATTTG | 64 |
| 11 | Beta-glucosidase 5 | gi|75285316 | CAGGGTTGCACACTGGATTACA | TCCGGAATCATAAGACCCAATT | 66 |
| 13 | Phosphoribulokinase | gi|115448091 | CCCCCCAAGATTTTTGTCATT | CCCTCACACGTTCATCAAACA | 58 |
| 48 | HSP70 | gi|115452223 | CAGATCACCGTGTGCTTCGA | TCTGCCCGGTGGTCTTGT | 76 |
| 53 | Putative 33kDa oxygen evolving protein of photosystem II | gi|115436780 | TCGACTCCTTCGCCTTCAA | CGGCTCCAGGCAGAACTTC | 59 |
|  | Tubulin | gi|218189 | TTGTCCTGCGCCTCCGT | GGCATAGGTATAATGAAGTCCAATGC | 71 |
